# Pan-European Study on the Prevalence of the Feline Leukaemia Virus Infection – Reported by the European Advisory Board on Cat Diseases (ABCD Europe)

**DOI:** 10.3390/v11110993

**Published:** 2019-10-29

**Authors:** Nadine Studer, Hans Lutz, Claude Saegerman, Enikö Gönczi, Marina L. Meli, Gianluca Boo, Katrin Hartmann, Margaret J. Hosie, Karin Moestl, Séverine Tasker, Sándor Belák, Albert Lloret, Corine Boucraut-Baralon, Herman F. Egberink, Maria-Grazia Pennisi, Uwe Truyen, Tadeusz Frymus, Etienne Thiry, Fulvio Marsilio, Diane Addie, Manfred Hochleithner, Filip Tkalec, Zsuzsanna Vizi, Anna Brunetti, Boyko Georgiev, Louisa F. Ludwig-Begall, Flurin Tschuor, Carmel T. Mooney, Catarina Eliasson, Janne Orro, Helle Johansen, Kirsi Juuti, Igor Krampl, Kaspars Kovalenko, Jakov Šengaut, Cristina Sobral, Petra Borska, Simona Kovaříková, Regina Hofmann-Lehmann

**Affiliations:** 1 Clinical Laboratory, Department of Clinical Diagnostics and Services, and Center for Clinical Studies, Vetsuisse Faculty, University of Zurich, 8057 Zurich, Switzerland; 2 Department of Infectious and Parasitic Diseases, Research Unit of Epidemiology and Risk Analysis Applied to Veterinary, Fundamental and Applied Research for Animal and Health (FARAH) Center, Faculty of Veterinary Medicine, University of Liège, B-4000 Liège, Belgium; 3 Department of Geography, University of Zurich, 8057 Zurich, Switzerland; 4 Clinic of Small Animal Medicine, Centre for Clinical Veterinary Medicine, LMU Munich, 80539 Munich, Germany; 5 MRC- University of Glasgow Centre for Virus Research, Glasgow G61 1QH, UK; 6 Institute of Virology, Department for Pathobiology, University of Veterinary Medicine, 1210 Vienna, Austria; 7 Bristol Veterinary School, University of Bristol, Bristol BS40 5DU, UK & Chief Medical Officer, Linnaeus Group, Shirley, Solihull B90 4BN, UK; 8 Swedish University of Agricultural Sciences (SLU), Department of Biomedical Sciences and Veterinary Public Health (BVF), 750 07 Uppsala, Sweden; 9 Fundació Hospital Clínic Veterinari, Universitat Autònoma de Barcelona, 08193 Bellaterra, Barcelona, Spain; 10 Scanelis laboratory, 31770 Colomiers, France; 11 University of Utrecht, Faculty of Veterinary Medicine, Department of Infectious Diseases and Immunology, 3584 CL Utrecht, Netherlands; 12 Dipartimento di Scienze Veterinarie, Università di Messina, 98168 Messina, Italy; 13 Institute of Animal Hygiene and Veterinary Public Health, University of Leipzig, 04103 Leipzig, Germany; 14 Department of Small Animal Diseases with Clinic, Faculty of Veterinary Medicine, Warsaw University of Life Sciences-SGGW, 02-787 Warsaw, Poland; 15 Veterinary Virology and Animal Viral Diseases, Department of Infectious and Parasitic Diseases, FARAH Research Centre, Faculty of Veterinary Medicine, Liège University, B-4000 Liège, Belgium; 16 Faculty of Veterinary Medicine, Università degli Studi di Teramo, 64100 Teramo, Italy; 17 Veterinary Diagnostic Services, School of Veterinary Medicine, College of Medical, Veterinary and Life Sciences, University of Glasgow, Glasgow G61 1QH, UK; 18 Tierklinik Strebersdorf, 1210 Vienna, Austria; 19 Veterinarska klinika Kreszinger, 10360 Sesvete, Zagreb, Croatia; 20 University of Veterinary Medicine, 1078 Budapest, Hungary; 21 School of Veterinary Medicine, University of Glasgow, Glasgow G61 1QH, UK; 22 Institute of Biology and Immunology of Reproduction, 1113 Sofia, Bulgaria; 23 Kleintierklinik BolligerTschuor AG, Fachtierärzte für Kleintiere, 4665 Oftringen – Zofingen, Switzerland; 24 School of Veterinary Medicine, University College Dublin, Belfield, Dublin 4, Ireland; 25 Jamaren - Swedish Veterinary Feline Study Group, 275 71 Lövestad, Sweden; 26 Loomakliinik, 51014 Tartu, Estonia; 27 Bygholm Dyrehospital, 8700 Horsens, Denmark; 28 CatVet Kissaklinikka, 00400 Helsinki, Finland; 29 Slovak Small Animal Veterinary Association, 821 02 Bratislava, Slovakia; 30 Faculty of Veterinary Medicine, Latvia University of Lifesciences and Technologies, LV-3004 Jelgava, Latvia; 31 Jakov Veterinary Centre, Gerosios Vilties g. 1, LT-03147 Vilnius, Lithuania; 32 Vetalmada, small animal clinic, 2800-052 Almada, Portugal; 33 Small Animal Emergency Clinic, 637 00 Brno-Jundrov, Czech Republic; 34 Department of Animal Protection, Welfare and Behavior, Faculty of Veterinary Hygiene and Ecology, University of Veterinary and Pharmaceutical Sciences Brno, 612 42 Brno, Czech Republic

**Keywords:** FeLV, retrovirus, prevalence, risk factors, protective factors, RT-qPCR, virus shedding, vaccination, gross domestic product at purchasing power parity per capita, veterinary sciences

## Abstract

Feline leukaemia virus (FeLV) is a retrovirus associated with fatal disease in progressively infected cats. While testing/removal and vaccination led to a decreased prevalence of FeLV, recently, this decrease has reportedly stagnated in some countries. This study aimed to prospectively determine the prevalence of FeLV viraemia in cats taken to veterinary facilities in 32 European countries. FeLV viral RNA was semiquantitatively detected in saliva, using RT-qPCR as a measure of viraemia. Risk and protective factors were assessed using an online questionnaire to report geographic, demographic, husbandry, FeLV vaccination, and clinical data. The overall prevalence of FeLV viraemia in cats visiting a veterinary facility, of which 10.4% were shelter and rescue cats, was 2.3% (141/6005; 95% CI: 2.0%–2.8%) with the highest prevalences in Portugal, Hungary, and Italy/Malta (5.7%–8.8%). Using multivariate analysis, seven risk factors (Southern Europe, male intact, 1–6 years of age, indoor and outdoor or outdoor-only living, living in a group of ≥5 cats, illness), and three protective factors (Northern Europe, Western Europe, pedigree cats) were identified. Using classification and regression tree (CART) analysis, the origin of cats in Europe, pedigree, and access to outdoors were important predictors of FeLV status. FeLV-infected sick cats shed more viral RNA than FeLV-infected healthy cats, and they suffered more frequently from anaemia, anorexia, and gingivitis/stomatitis than uninfected sick cats. Most cats had never been FeLV-vaccinated; vaccination rates were indirectly associated with the gross domestic product (GDP) per capita. In conclusion, we identified countries where FeLV was undetectable, demonstrating that the infection can be eradicated and highlighting those regions where awareness and prevention should be increased.

## 1. Introduction

Feline leukaemia virus (FeLV) is a gammaretrovirus that infects domestic cats and closely related wild felids worldwide. Since the first description of the virus in 1964 in feline lymphoma tissue [1], increasing knowledge has been gained concerning the pathogenesis, diagnosis, and treatment of the infection [2,3]. FeLV infection can lead to fatal diseases in cats with progressive infection [4,5,6,7,8,9]. Testing and eradication programs and the introduction of effective vaccines led to a decrease in FeLV prevalence in many countries in the last 30 years [7,10,11,12]. However, more recently, the decrease of the FeLV prevalence has stagnated [13,14,15]. In other countries, such as Denmark, FeLV is rarely detected nowadays [16]. For the USA, Canada, and Australia, large prevalence studies have been published [10,13,17,18,19]. There have also been some recent studies on the prevalence of FeLV in single European countries [15,20,21,22]. However, there has been no pan-European study determining the current FeLV prevalence in domestic cats. FeLV prevalence can vary considerably depending on the composition of the investigated cat population; feral or stray cats versus privately owned cats, cats in shelters or from breeders, clinically healthy cats or sick cats [17,23,24,25]. In addition, preselection of the samples has an influence; e.g., if samples are obtained only from cats suspected of being infected with FeLV. A recent meta-analysis [26] suggested an indirect relation between FeLV prevalence and the yearly gross domestic product (GDP) per capita using purchasing power parity (PPP) [27], i.e., the total value of all the goods and services produced by a country in a particular year, divided by the number of people living there and corrected for purchasing power using a “basket of goods”. However, no FeLV prevalence data were available for many European countries, or the data were obtained several years ago.

The aim of the present study was to determine the current prevalence of FeLV viraemia in cats that visit a veterinary facility. The study protocol included cats from 32 European countries in the survey. In order to avoid any preselection of the cats (apart from visiting a veterinary facility), 10 animals arriving consecutively at each facility were tested independently of the reason for the veterinary appointment. Risk and protective factors for FeLV infection were determined using the demographic, husbandry, clinical as well as FeLV vaccination history data from each cat. Furthermore, using our data, we tested the recently postulated hypothesis of a correlation between the FeLV prevalence and the GDP in European countries, and extended the analysis to test also for a potential correlation with FeLV vaccination rates. For minimally invasive sample collection and to avoid bias by requesting blood collection, saliva swabs were collected and analysed by RT-qPCR; the detection of viral RNA by RT-qPCR in saliva was shown to be an excellent measure of FeLV viraemia [28].

## 2. Materials and Methods

### 2.1. Design of the Prevalence Study

The study design included 920 European veterinary facilities in 32 countries; for organizational/financial reasons, some small countries were grouped into country groups, resulting in 23 countries/country groups (Table 1). The countries/country groups were classified into “Eastern Europe”, “Northern Europe”, “Southern Europe”, and “Western Europe” according to the United Nations geoscheme [29]. According to the policy coordinator of the European Union Commission (Directorate-General for Environment, Unit Chemicals), no animal testing permit was required for this study, mainly because of the non-invasive method of sample collection, sample collection occurring during regular visits to veterinary practices, and the handling of the cats by veterinary professionals. In Switzerland, the study was officially approved by the veterinary offices of the Swiss cantons (approval number: ZH 121/16, issued 11 August 2016) and conducted according to Swiss laws. Written informed consent was obtained from each cat owner by the participating veterinarians in all countries. The sample collection was conducted from September 2016 to March 2017.

### 2.2. Sample Collection for the Prevalence Study

Forty veterinarians were involved per country/country group; they were instructed to collect saliva swabs from 10 cats during consecutive veterinary consultations. This resulted in an intended maximal number of 400 samples per country/country group and a total maximal intended number of 9200 samples. In the country groups, the number of samples per single country was chosen in relation to the human population size of the respective country [29], since for many countries, reliable estimates of cat populations were unavailable. Whenever possible, the veterinarians were chosen from different areas within a country.

Participating veterinarians were provided (by priority mail) with 10 labeled screw-cap tubes (1.5 mL, Sarstedt, Nümbrecht, Germany) filled with 300 µL of RNA shield (Zymo Research Europe GmbH, Freiburg im Breisgau, Germany), 10 cotton swabs with plastic shafts (M-Budget, Migros Genossenschafts-Bund, Switzerland), an instruction sheet that described the proper swabbing procedure, informed consent forms to be signed by the cat owners, customs declaration, import permits, and prepaid return address labels. The RNA shield was provided to ensure biological safety during the shipment of the saliva samples, since rabies is encountered in some participating countries [30,31], and also to increase the stability of FeLV viral RNA. The veterinarians were asked to sample 10 cats during consecutive appointments, regardless of the animal’s age, sex, vaccination status, health status, or reason for the veterinary consultation. Only one cat per home, breeder, or shelter was to be sampled. The swab was to be rubbed gently along the cheek pouches and under the tongue of the cat, placed in the tube, and the external tip of the swab was removed prior to closing the tube. The samples were shipped by postal mail at ambient temperature.

### 2.3. Data Collection for the Prevalence Study

For each sampled cat, an online questionnaire was completed by the sampling veterinarian. The questionnaire was available in 18 languages and included 19 questions concerning geographic data (country and postal code of cat owner), sample identification (identification number, name of the veterinary practice, cat and cat owner, date of collection), demographic data (age, sex, reproductive status and breed of the cat), husbandry data (type of husbandry, such as private home, cat breeder, animal shelter, rescue cat, number of cats per household, and outdoor access) data on FeLV vaccination history, and the results of the physical examination (healthy versus sick and, if sick, the major clinical problem) (for details, see Appendix A Table A1). The physical examination was performed during regular visits of the cat by the attending veterinarian, who assessed the health condition of the cat based on his/her professional experience. A similar questionnaire has been used in previous studies [32,33,34].

### 2.4. Sample Preparation and Molecular Analysis

**Figure A1:**
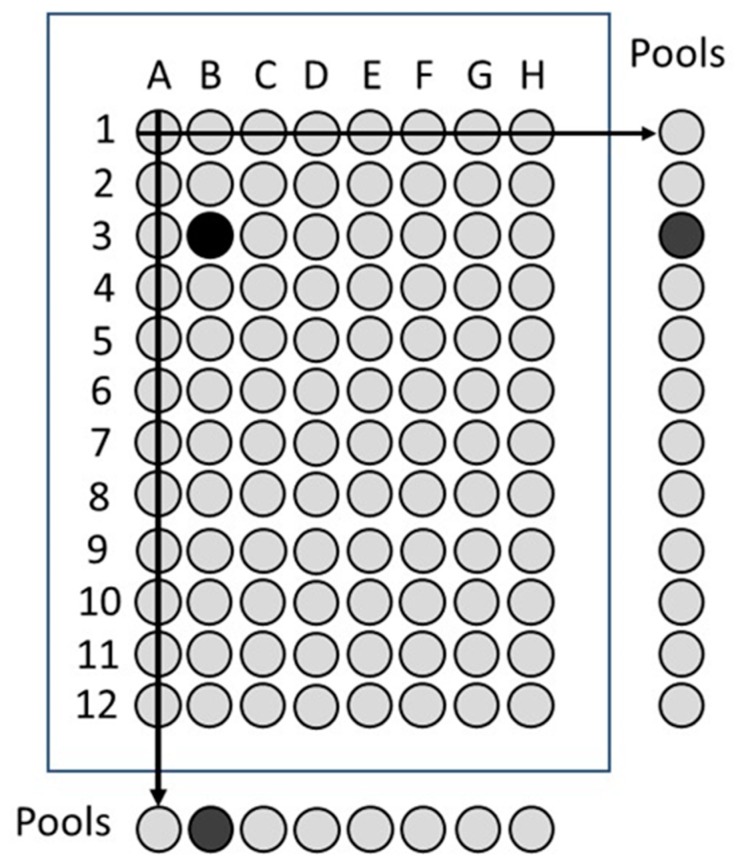
Schema of the pooling of the saliva samples: 96 samples (A1 to H12) were pooled to 20 pools. Each pool consisted of 200 µL; either 8 × 25 µL (horizontal pooling) or 12 × 16.7 µL (vertical pools). After pooling, each sample was present in two pools. Example: If a sample is FeLV-positive (black circle, B3), two pools resulted in positive RT-PCR results.

Samples were processed upon receipt in the laboratory as described previously [28,34,35,36]. The tubes were vortexed and put on a shaking incubator at 42 °C for 10 min to resuspend the sample. Subsequent sample preparation was performed under sterile conditions in a laminar flow cabinet. After centrifugation at 8000× *g* for 1 min to remove any liquid from the inside of the lid, the swabs were inverted using a pair of sterilized tweezers and centrifuged again to recover the liquid (freed from the cotton part of the swab) in the bottom of the tube. The swabs were removed, and the liquid sample material was stored at −80 °C until further use. Subsequently, the liquid samples were pooled (Pipetting robot CAS-1200, LTF Labortechnik GmbH & Co. KG, Wasserburg, Germany) such that up to 96 samples were combined in 20 pools and the material from each sample was present in two pools (for details, see Appendix A Figure A1). Total nucleic acid (TNA) was extracted from the sample pools using the MagNA Pure LC Total Nucleic Acid Kit - High Performance and the MagNA Pure LC instrument (Roche Diagnostics, Mannheim, Germany), following the instructions of the manufacturer, with an elution volume of 90 μL. Two negative controls of phosphate-buffered saline (PBS) were concurrently prepared with each batch of samples to monitor for cross-contamination.

FeLV viral RNA was detected using 5 μL of TNA, and a previously described real-time TaqMan FeLV RT-qPCR [37] on an ABI PRISM 7500 Fast Sequence Detection System (Applied Biosystems, Foster City, USA) with some modifications. Briefly, the 25-μL RT-qPCR reaction contained 12.5 μL 2× RT-qPCR Buffer, 1 µL 25× RT-qPCR Enzyme Mix (AgPath-IDTM One-Step RT-qPCR Reagents, Thermo Fisher Scientific), a final concentration of 900 nM of forward primer (FeLV_U3_exo_f; 5’AAC AGC AGA AGT TTC AAG GCC 3’; 21 bp), 300 nM of reverse primer (FeLV_U3_exo_r; 5’TTA TAG CAG AAA GCG CGC G3’; 19 bp), and 200 nM of fluorogenic probe (exoFeLV-U3-probe; 5’-FAM-CCA GCA GTC TCC AGG CTC CCC A-TAMRA 3’; 22 bp). All oligonucleotides were synthetized by Microsynth AG (Balgach, Switzerland). The temperature profile was 10 min at 45 °C, followed by 10 min at 95 °C and 40 cycles of 15 s at 95 °C, followed by 45 s at 60 °C. Each PCR run was performed together with positive (RNA standard template) [37] and negative controls (PBS).

The pooling scheme allowed the identification of the individual samples that could have contributed to the positive pool results. From all these single samples, TNA was extracted from 50 μL of original liquid sample material, and FeLV real-time RT-qPCR was performed as described above. The FeLV input copy numbers in the single samples were determined by co-amplifying 10-fold serial dilutions of an RNA standard template as described [37]. All further analyses were conducted with the FeLV RT-qPCR results of the individual samples/cats.

### 2.5. Pre-Experiment

The stability of FeLV in the RNA shield was tested in a pre-experiment using cell culture supernatant from FeLV-infected FL-74 cells. Cell culture supernatant was diluted in PBS to reach a FeLV copy number concentration that corresponded to that in the saliva of viraemic cats (threshold cycle values of approximately 22–24) [37,38]. Aliquots of diluted cell culture supernatant were stored at room temperature for 7, 14, 21, 28, 50, and 60 days and at 37 °C for 2, 3, 5, 7 and 14 days, respectively. After incubation, TNA was extracted and analysed by FeLV real-time RT-qPCR as described above.

### 2.6. Statistical Analysis

#### 2.6.1. Descriptive Analysis

The parameters of all cats were compiled and analysed using Excel (Microsoft, Wallisellen, Switzerland) and GraphPad Prism software (GraphPad, San Diego, CA). FeLV prevalence (viraemia) in sick and healthy cats and frequencies of FeLV viral RNA loads, classified as high and low loads (> or ≤10^6^ copies/PCR reaction), were analysed using the chi-square test (p_Chi_) or Fisher’s exact test (p_F_). Visualization of the data was performed by geocoding the data of the cat owners using RStudio (version 1.1.383, RStudio Team, 2016, Integrated Development for R. RStudio, Inc., Boston, MA). The following packages were used: ggmap [39], cartography [40], and spatialEco [41].

#### 2.6.2. Regression Analysis

The relationships between firstly the percentage of FeLV prevalence and GDP per capita at PPP and secondly the percentage of FeLV vaccination rate and GDP per capita at PPP were first evaluated using the Pearson correlation coefficient (parametric hypothesis concerning the relation) as well as the Spearman correlation coefficient (non-parametric hypothesis concerning the relation). The GDP per capita converted to US dollars using PPP was assigned to each country/country group using the International Monetary Fund World Economic Outlook (October 2018) [27]. For significant correlation, regression was applied to predict the relationship between the variables investigated with a 95% confidence interval. In addition, the normality of the distribution of studentized residuals was tested in order to validate the regression model. 

To identify possible risk and protective factors associated with FeLV infection, the responses to the questionnaire were encoded and merged with the RT-qPCR results (classified as positive or negative) of each cat. First, a univariate analysis was conducted and odds ratios (OR) with 95% confidence intervals (95% CI) were attributed to each variable. Next, a multivariate logistic regression analysis was performed using variables with *p*-values < 0.20. In some cases, the use of the Firth logit method allowed an inference of ORs and CIs when complete separation (zero cells) occurred [42]. Backward stepwise logistic regression was used to exclude progressively variables having the highest *p*-value [43]. In that final model, all pairwise interactions between variables, if biologically relevant, were examined for significance. The goodness of fit was assessed through a multivariate logistic regression using the Hosmer–Lemeshow goodness-of-fit test [43]. Statistical analyses were carried out in STATA/SE 14.2 (StataCorp, College Station, TX, USA). The limit of statistical significance of the tests performed was defined as 0.05.

#### 2.6.3. Classification Tree Analysis

Classification and regression tree (CART) analysis [44,45] had been evaluated previously in medical science [46,47]. Further details and explanations of this CART analysis are available in previous veterinary articles, including a prevalence study of feline retroviruses [48,49,50,51,52]. Classification trees are trained by passing data down from a root node to leaves. The data is repeatedly split according to predictor variables so that child nodes are more “pure” (i.e., homogeneous) in terms of the outcome variable (i.e., FeLV status). In this study, a classification tree analysis was based on the subdivision of the data set into randomly selected and approximately equal parts, with each “part” containing a similar distribution of data from the populations of interest (i.e., positive versus negative results for FeLV). Then, the analysis used the first nine parts of the data, constructing the largest possible tree, and used the remaining 1/10th of the data to obtain initial estimates of the error rate of the selected subtree. The process was repeated, using different combinations of the nine remaining data subsets and a different 1/10th data subset to test the resulting tree. This process was repeated until each 1/10th data subset had been used to test a tree that had been grown using 9/10ths of the data. Then, the results of the 10 mini-tests were combined to calculate the error rates for trees of each possible size; these error rates were applied to prune the tree that had been grown using the entire data set. In order to test the diagnostic power of the final decision tree generated, a receiver operating characteristic (ROC) curve was used for both the data set that was used to build the tree (learning data set) and the data set that was used to test the adequacy of the tree to the data (testing data set). This complex process resulted in a set of fairly reliable estimates of the independent predictive accuracy of the tree [53]. Classification tree analysis was carried out in a Salford Predictive Modeler (Salford Systems, San Diego, CA, USA).

## 3. Results

### 3.1. Pre-Experiment

When testing the stability of FeLV in RNA shield buffer at room temperature for up to 60 days and at 37 °C for up to 14 days, no significant loss of FeLV viral RNA was observed (<10-fold decrease).

### 3.2. Pan-European Prevalence Study

#### 3.2.1. Sample Size and Return Rate

**Figure 1:**
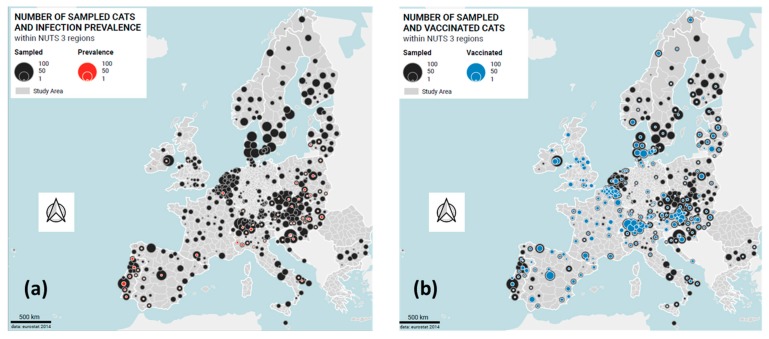
Origin and FeLV infection and vaccination status of the cats visiting veterinarians in the 30 European countries. (**a**) FeLV viraemia: black: all cats; red: FeLV-positive cats. (**b**) FeLV vaccination status: black: all cats; blue: FeLV vaccinated cats. The size of the circle represents the number of cats.

With the support of the country representatives, 861 veterinary facilities were enrolled, 93.6% of the initially intended total of 920 facilities (Table 1). Subsequently, 6720 samples (78.0% of the 8610 shipped tubes) were returned to the laboratory; these samples originated from 30 of the 32 originally included countries (Table 1; Figure 1a).

In Greece, the recruitment of veterinarians was unsuccessful, while in Romania samples were collected, but owing to problems with shipping none were received by the laboratory in Switzerland. Data were provided for 6005 of the 6720 returned samples using the online questionnaire (69.7%). Subsequently, these 6005 samples accompanied with a complete data set were included in the further analysis.

#### 3.2.2. Sample Characteristics

**Figure 2:**
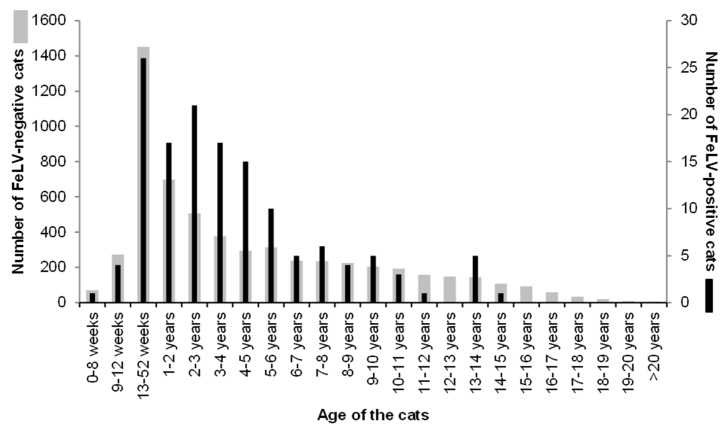
Age distribution of the cats: grey: FeLV-negative cats (left axis), black: FeLV-positive cats (right axis). Cats aged from one to six years were significantly more frequently FeLV-positive than younger or older cats (*p*_F_ < 0.0001; see also Table 3).

Approximately two-thirds of the 6005 cats were clinically healthy (*n* = 4060, 67.6%); 1945 cats were sick at the time of veterinary consultation (32.4%; Table 2). The age distribution of the 6005 cats is shown in Figure 2. Overall, the sex and reproductive status was known for 5886 cats (Table 2); in 119 cats, either the sex or the reproductive status was listed as “not sure”. Approximately two-thirds of the cats had been spayed or castrated (68.6%); approximately one-third was sexually intact (29.4%). 

Pedigree status was known for 5948 cats; most were non-pedigree cats (*n* = 5156; 85.9%). Of the 792 pedigree cats, 118 were British shorthairs, 110 were Persians, 99 were Maine Coons, 51 were Ragdolls, 42 were Siamese, 35 were Norwegian Forest cats, and 30 were Birman cats; from the remaining breeds, fewer than 30 cats had been included. The large majority of the cats were kept in private homes (*n* = 5151; 85.9%); much less frequently sampled were rescue cats and cats from breeders or animal shelters (Table 2). The information concerning group housing or being a single cat was known for 5753 cats, of which 3373 cats lived in multicat environments (56.2%), and 2380 were kept as single cats (39.6%; Table 2). Approximately half of the cats in multicat environments (*n* = 1670; 49.5%) had only one companion cat. Most cats had outdoor access (*n* = 3633; 60.5%; Table 2); these cats could be further divided into cats that lived indoors and had outdoor access (*n* = 3245; 54.0%) and cats that always lived outdoors (*n* = 388 cats; 6.5%). Finally, approximately one-third of all cats lived strictly indoors (*n* = 2193; 36.5%). Information concerning FeLV vaccination status and the timepoint of the most recent FeLV vaccination was available for 5400 cats (Table 2 and Figure 1b).

#### 3.2.3. Prevalence of FeLV Viraemia

**Figure 3:**
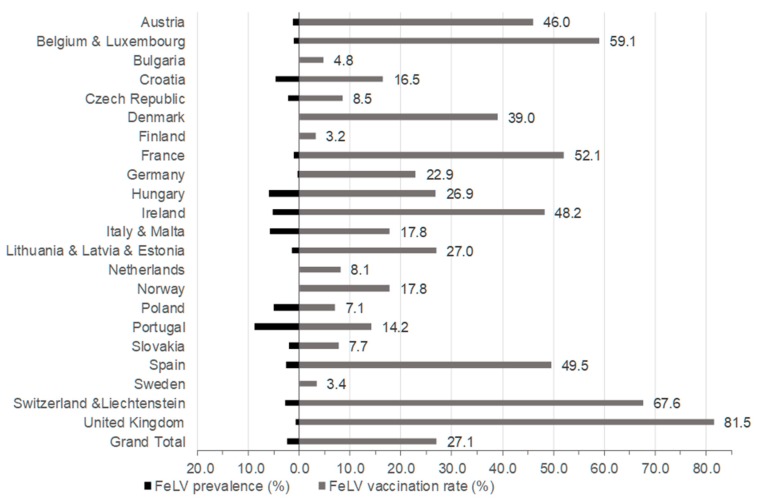
FeLV prevalence (on the left) and FeLV vaccination rates (on the right) in the different countries/country groups. In the FeLV vaccination rates, cats with unknown vaccination status were not included (~10% of the cats).

Of the 6005 samples accompanied with data from the questionnaire, 141 samples tested FeLV-positive by real-time RT-qPCR (2.3%; 95% CI: 2.0–2.8; Table 1) from saliva. The individual samples that tested RT-qPCR positive had been identified from the sample pools that had tested positive. At least one individual sample tested FeLV RT-qPCR positive in each set of samples that had contributed to a positive pool. The countries/country groups with the highest prevalence of FeLV viraemia were Portugal (8.8%; 95% CI: 6.2–12.3%), Hungary (5.9%; 95% CI: 3.5–9.9%), and Italy and Malta (5.7%; 95% CI: 3.7–8.7%; Table 1 and Figure 3). None of the tested samples was FeLV-positive in the following countries: Denmark, Finland, Norway, Sweden, Latvia, Estonia, Scotland, Wales, Bulgaria, Malta, Luxembourg, and the Netherlands. Moreover, only one FeLV-positive sample was obtained from Germany and England (Table 1 and Figure 3). 

The prevalence of FeLV viraemia in sick cats was 3.9% (75 of 1945 cats; 95% CI: 3.0–4.8%) and in healthy cats 1.6% (66 of 4060 cats; 95% CI: 1.3–2.1%; Table 2 and Table 3). Sick cats were significantly more frequently FeLV-positive than healthy cats (*p*_F_ < 0.0001; OR 2.4, 95% CI: 1.7–3.4%; Table 3). When the age distribution of the FeLV-positive and FeLV-negative cats was inspected graphically (Figure 2), it was evident that cats aged one to six years were more frequently FeLV-positive than cats of other ages. Thus, cats were categorized accordingly for further statistical analysis: cats less than one year of age; cats between one and six years of age; and cats older than six years.

#### 3.2.4. FeLV Vaccination Status

For 5400 cats, the veterinarians provided information concerning FeLV vaccination status. Amongst these cats, 3938 had never been vaccinated against FeLV (65.6% of all cats); 1462 cats were known to have been vaccinated at least once (24.3% of all cats; Table 2 and Figure 1b). Young cats (<1 year of age) were less frequently vaccinated (18.7%) compared to older cats (1 to ≤6 years: 25.1%; >6 years: 28.8; *p*_Chi_ < 0.0001). Among the 141 FeLV-positive cats, the vaccination status was known for 120 cats; amongst those cats, 14 (11.7%) had been vaccinated at some timepoint. FeLV-positive cats had been vaccinated significantly less frequently against FeLV compared to FeLV-negative cats (*p*_F_ < 0.0001). Vaccination rates varied considerably, ranging from 3.2% and 3.4% in Finland and Sweden, respectively, to 67.6% and 81.5% in Switzerland and Liechtenstein and the UK, respectively (Figure 3). The vaccination rates in the three countries/country groups with the highest FeLV prevalence, namely Portugal, Hungary, and Italy and Malta, were rather low, at 14.2%, 26.9%, and 17.8%, respectively (Figure 3).

#### 3.2.5. Regression Analysis

First, a univariate regression analysis was performed (Table 3). Then, all variables with *p*-values < 0.20 in the univariate analysis were entered in a multivariate analysis. A backward, stepwise strategy was used to obtain a final multivariate logistic regression analysis model (Table 4). Following the multivariate analysis, seven risk factors (origin of cats from Southern Europe, male intact, one to six years of age, indoor and outdoor or outdoor-only living, living in a group of ≥5 cats, sick cats), and three protective factors (origin from Northern Europe, origin from Western Europe, and pedigree cats) were identified (*p* < 0.05). There was a tendency that cats vaccinated against FeLV during the year prior to sampling were less frequently infected (8/943) compared to cats that had never been vaccinated (106/3938; *p* = 0.06; Table 2 and Table 4). The Hosmer–Lemeshow goodness-of-fit test (chi-square (8 degrees of freedom) = 10.85; *p*-value = 0.21) indicated that the final model fit the data well.

**Figure 4:**
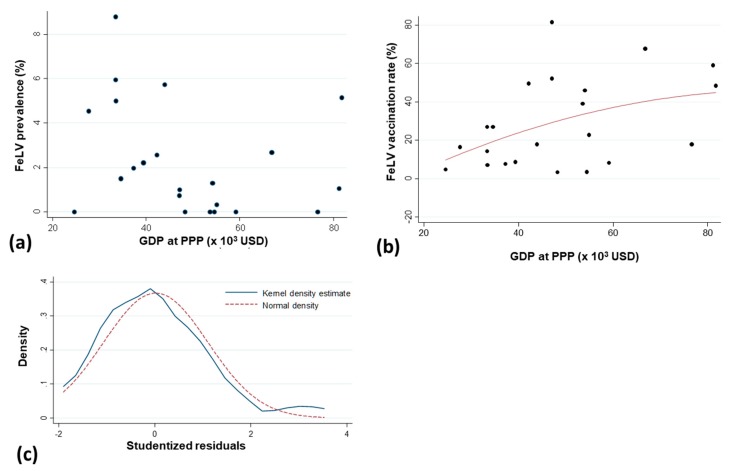
Relation between FeLV prevalence or FeLV vaccination rate and the gross domestic product (GDP) per capita purchasing power parity (PPP). (**a**) Relation between FeLV prevalence and GDP per capita using PPP in US dollars (USD); (**b**) Linear relation between FeLV vaccination rate and GDP per capita using PPP in USD (with the black points, the observed values; the line, the linear relation between the FeLV vaccination rate and the GDP per capita PPP and its 95% confidence interval, with the following equation: FeLV_vacc = −0.9698946 + (0.6052006 * GDP); (**c**) Density of the studentized residuals with the kernel density estimate and the normal density as reference. Kernel = epanechnikov, bandwidth = 0.4433.

When testing the relationship between the GDP per capita at PPP and the percentage of FeLV prevalence, neither parametric nor non-parametric significant correlations were found between GDP and FeLV prevalence (Pearson correlation coefficient = –0.29 with *p* = 0.18 and Spearman rank correlation coefficient = –0.37 with *p* = 0.09) (Figure 4a). However, there was a significant linear correlation between GDP at capita PPP and the FeLV vaccination rate (*p* = 0.045; Figure 4b). The normality of the distribution of studentized residuals was acceptable; see Figure 4c.

#### 3.2.6. Classification Tree Analysis

**Figure 5:**
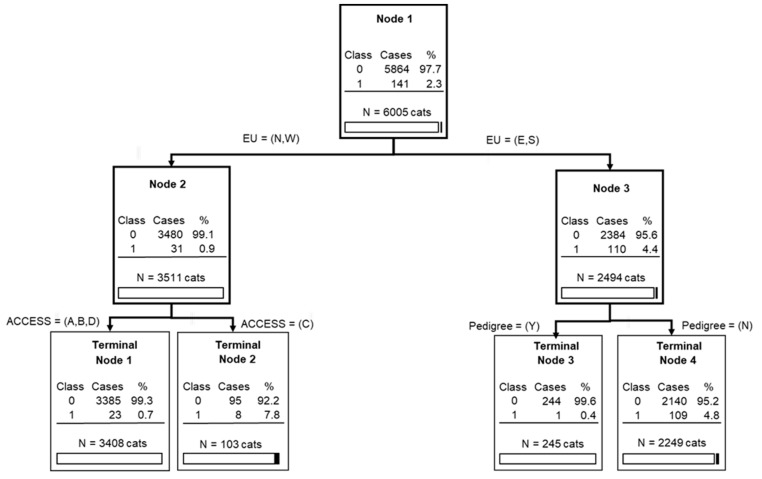
Classification tree analysis for FeLV viraemia. Legend: Class: 0 = negative for FeLV; 1 = positive for FeLV. EU = Europe: N, North; W, West; E, East; S, South. Access: A, Indoor only; B, Indoor and outdoor; C, Outdoor only; D, Not sure. Pedigree: Y, Yes; N, No.

According to the classification tree analysis (Figure 5), the origin of cats within Europe (discriminatory power (DP) of 100, with a scale between 0 and 100), having a pedigree (DP = 55.60), and living outdoors only (DP = 29.08) were the three important predictors (or splitters) regarding a cat’s FeLV status, with a relatively good tree sensitivity and specificity: 82.98% (95% CI: 75.74–88.78) and 61.89% (95% CI: 60.63–63.13), respectively. The areas under the ROC curve for the learning data and the test data set were 0.73 and 0.69, respectively. These values indicate the potential of the proposed tree to discriminate between the diagnoses (FeLV-negative versus FeLV-positive).

#### 3.2.7. FeLV Viral RNA Loads in Saliva

**Figure 6:**
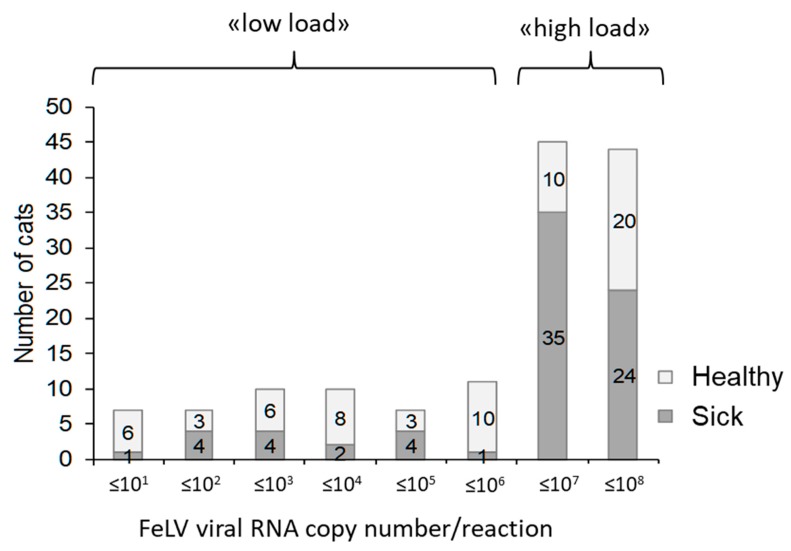
Distribution of salivary FeLV viral RNA loads in FeLV-positive healthy and sick cats. Loads are given as copies per PCR reaction conducted with 5 μL of total nucleic acid extracted from 100 μL of liquid from saliva swab samples.

Viral load determination in saliva samples was semiquantitative, since the volume of collected saliva and the biological dilution of the saliva were unknown. Moreover, each sample had been stored in 300 µL of RNA shield that stabilized, but also further diluted, the saliva sample. Nevertheless, by employing quantitative FeLV real-time RT-qPCR to determine FeLV viral RNA loads in the available samples, between three copies and 6.9 × 10^7^ copies per PCR reaction were detected using 5 μL of input TNA. Approximately two-thirds (63%) of the samples contained high FeLV viral RNA loads with >10^6^ copies per PCR reaction (Figure 6). Samples from sick FeLV-infected cats contained more frequently high FeLV viral RNA loads (>10^6^ copies per PCR reaction; 59 of 75 cats; 79%) than samples from healthy FeLV-infected cats (30 of 66 cats; 45%; *p*_F_ < 0.0001; OR 4.4, 95% CI: 2.1–9.2). However, healthy FeLV-positive cats also shed high viral RNA copy numbers in their saliva; see Figure 6.

#### 3.2.8. Clinical Signs Associated with FeLV Infection

The major clinical problems (inclusion optional in the questionnaire) that were reported in the 75 FeLV-positive sick cats included upper respiratory tract diseases (URTD: “cat flu”, nasal or ocular discharge, conjunctivitis, sneezing; *n* = 11), gingivitis and/or stomatitis (*n* = 11), anaemia (*n* = 8), anorexia (*n* = 6), tumour (lymphoma, rhabdomyosarcoma, liver tumour; *n* = 4), diarrhoea (*n* = 3), and abscess (*n* = 3). Less frequently reported were fever, apathy, renal failure, jaundice, and various other clinical signs. FeLV-positive sick cats more frequently displayed anaemia (OR 9.6, 95% CI: 4.4–21.4; *p*_F_ < 0.0001), anorexia (OR 3.5, 95% CI: 1.6–8.3; *p*_F_ = 0.0122) and gingivitis and/or stomatitis (OR 2.5, 95% CI: 1.3–4.8; *p*_F_ = 0.0152) than FeLV-negative sick cats (Table 5).

## 4. Discussion

The aim of the present study was to evaluate the prevalence and significance of FeLV infection in cats taken to veterinarians in Europe. The investigation was conducted prospectively and at a pan-European level; it is the first study of its kind. The study aimed to include 32 countries, of which 30 successfully contributed samples. To the best of the authors’ knowledge, this study provides the first FeLV prevalence data for some European countries, i.e., Latvia, Lithuania, Bulgaria, Hungary, and Croatia, and current data for some countries without recent information on FeLV prevalence, i.e., France, Finland, Czech Republic, and the Netherlands. By using saliva swabs rather than an invasive method such as blood collection, samples could be collected from all cats visiting the veterinarians, thereby reducing the bias of selecting only cats that were easy to handle or that were presented because of illness and required blood collection for routine diagnostics. Saliva samples collected using buccal swabs were analysed for FeLV viral RNA using RT-qPCR. There is an almost perfect agreement between the shedding of FeLV viral RNA in saliva and the presence of viraemia, as reported previously [28,35]; therefore, the results obtained were comparable with those of other studies measuring free FeLV p27 antigen in blood samples. FeLV antigenemia is in most—but not all—cats a measure for FeLV viraemia [54]. Discrepancies can be found, particularly in the early stages of FeLV infection and in cats with a focal FeLV infection [55,56,57]. The saliva swabs had to be transported in an RNA shield buffer to ensure biological safety during shipment, since rabies is encountered in some of the countries included in the study [30,31]. At the same time, the buffer increased the stability of the FeLV viral RNA.

The overall prevalence of FeLV viraemia of 2.3% (95% CI: 2.0–2.8%) found in cats presented to veterinary facilities in Europe in the present study is within the range of other similar reports, where both healthy and sick cats from different environmental conditions have been tested [10,15,23]. In a large prevalence study conducted in the United States and Canada in 2010, the overall prevalence was somewhat higher, at 3.1% (95% CI: 3.0–3.3%), when testing for free FeLV p27 antigen [13]. However, the two surveys are difficult to compare. In the latter investigation, the samples were preselected; they had been collected by veterinarians and by staff at animal shelters with the intention of testing the cats for FeLV. In contrast, in the current study, all cats visiting European veterinary facilities were tested. Notably, the prevalence reported in the present study still might not reflect the overall FeLV prevalence in all cats within the countries included, although a number of stray and rescue cats had been tested also in the present study. Many cats, in particular those at risk of FeLV infection, might never visit a veterinary facility and thus would evade testing.

The prevalence of FeLV viraemia, as estimated by the present study, differed significantly in cats living in different regions of Europe. The investigations revealed that cats in Northern and Western Europe were at a lower risk of being FeLV-infected, while cats living in Southern European countries were at a much greater risk. In some Northern European countries, FeLV was even undetectable, i.e., Denmark, Finland, Sweden, and Norway. Remarkably, FeLV is a reportable disease in Sweden, and infections are registered by the Board of Agriculture (Jorbruksverket). FeLV is an unusual infection: in Sweden, six and seven cases were reported in 2015 and 2016, respectively [58]. In Denmark, FeLV prevalence was already low in 2010 in two out of three groups of stray cats and client-owned cats (0.8%; 0–0.9%), and a decrease of FeLV prevalence was reported in the third group; however, no detailed information was provided on FeLV tests or testing criteria [16]. In Finland, FeLV prevalence was also shown to be very low (1.0%) in 1990 in the Helsinki area in 196 stray cats [59]. Estonia, Latvia, and Lithuania are also Northern European countries [29]. Of these Baltic countries FeLV-positive cats were found only in Lithuania, the southernmost of these three countries (four positives of 143 cats tested; 2.8%). In Estonia, there has been one report from 173 shelter cats tested in 2014/2015, in which three cats tested positive for FeLV p27 antigen (1.7%) [60]; currently, there is one Estonian clinical case under investigation that tested positive for FeLV antigen; FeLV infection was confirmed by both virus isolation and provirus PCR (personal communication MJH and Olga Sjatkovskaja). The FeLV prevalence in the UK was low in the present study, with just one viraemic cat amongst 119 returned samples in England and no positives in Scotland and Wales (overall 0.7%); however, the return rate of samples was the lowest in the present study for the UK and Ireland compared to the other countries. An earlier study in the UK reported a high prevalence of FeLV in sick cats suspected of having FeLV or feline immunodeficiency virus (FIV) infections (18%) as well as in young healthy cats (5%) [24], but two more recent studies reported also a decreased FeLV prevalence in the UK: 3.5% of 517 stray cats in the Birmingham area (1.4% of the healthy cats; 6.9% of the sick cats) in 1997 were FeLV-infected, and 2.3% of the cats tested positive in two rehoming centres in the midlands and Eastern part of England in 2011/12 [61,62].

In some of the Northern European countries, where FeLV is an unusual infection or has not been reported for many years, it was observed that veterinarians vaccinated only infrequently against FeLV: the vaccination rate in Finland and Sweden was 3.2% and 3.4%, respectively (Figure 3). These low vaccination rates seem reasonable, since the infection risk is low, as long as no new FeLV cases are introduced. In contrast, 39.0% of the cats in Denmark were vaccinated against FeLV, which exceeds the European average (27.1%; Figure 3). Considering the very low FeLV infection risk for cats in Denmark, any vaccination strategy against FeLV might have to be reconsidered, taking into account the relative risk-to-benefit ratio for any subcutaneous injection [63]. The vaccination rate was even higher in the United Kingdom, where 81.5% of the cats in the present study had been FeLV vaccinated, and only 0.7% of the cats were FeLV-positive. Thus, veterinarians in the United Kingdom should be advised to vaccinate more selectively against FeLV by identifying those cats that might currently, or in the future, be at risk of FeLV infection.

A higher FeLV prevalence among the Northern European countries was found in Ireland (5.1%). This was higher than the prevalence reported previously in a study investigating 112 client-owned and stray cats in the Dublin area in 2007/2008 (1.8%) [64]. However, among the seven FeLV-positive cats in Ireland, five cats had undergone appointments at only two veterinary facilities in Galway and Kerry county, respectively. This suggests that there might be some geographic differences or regional FeLV hotspots within Ireland; this might also be the case in other countries. 

Amongst the Western European countries, no FeLV-infected cats were identified in the Netherlands in the current study: none of 356 cats tested FeLV RT-qPCR positive. In 1974, the prevalence of FeLV in the Netherlands was 9%, but it decreased to 3% by 1986 [11]. This success was attributed to the “test and removal program” that identified cats that carried FeLV and prevented their contact with uninfected cats [11]. In the present study, only 8.1% of the tested cats were vaccinated against FeLV in the Netherlands; this is in accordance with previous reports. Nonetheless, none of the cats in the Netherlands tested FeLV-positive. This highlights the importance of testing for maintaining an infection-free status, even in the absence of vaccination. A significant decrease in FeLV prevalence had also been reported in Switzerland: in 1990, 13.0% of sick cats were FeLV-positive [32] and a decrease to 3.1% in 2003 was demonstrated in a similar population of cats, using similar FeLV detection methods [15]. However, thereafter in Switzerland, the decrease in the FeLV prevalence stagnated; the most recent data from 2013–2016 reported a prevalence of 2.0% [15], which is similar to the prevalence found for Switzerland and Liechtenstein in the current study (2.7%). It is not clear why the FeLV prevalence did not decrease further in recent years; the FeLV vaccination rate reported in the present study was good, with 67.6% in Switzerland and Liechtenstein. However, not all cats at risk of FeLV infection appear to have been identified and FeLV vaccinated in time. In the other Western European countries—Germany, France, Austria, Belgium, and Luxemburg—the prevalence of FeLV (0.3–1.3%) was below the overall European prevalence (2.3%) and lower than that reported some years ago, e.g., in urban stray cats in the Belgic city of Ghent (1998–2000: 3.8%) [65], or in blood samples submitted for FeLV testing from owned cats in Austria (1996–2011: 5.6%) [22], but similar to a more recent study in Belgian stray cats sampled by veterinarians through a trap–neuter program (2010–2012: 0.7%) [52]. In Germany, the current data extends the decreasing trend observed between 1993 and 2002 [14].

Living in the Southern European countries was associated with a higher risk of FeLV infection (prevalence 2.6–8.8%). This is in accordance with a report from Portugal, where FeLV prevalence in stray cats in Lisbon was 5.7% in 2013/2014 [66]. For Italy, the most recent information about FeLV prevalence comes from a nationwide survey about *Leishmania infantum* infection, where 2659 leftover feline blood samples were tested also for retroviral DNA, and FeLV proviral DNA was recorded in 4.8% of cats, but unfortunately no regional or clinical data is available for these cats [67]. The present study demonstrated FeLV infections particularly in some Northern areas and in the Apulia region (Figure 1A). In previous reports, 3.8% and 6.1%, respectively, of stray cats in urban and rural colonies in northern Italy, Lombardy, tested FeLV antigen-positive (2008–2010 and 2014, respectively) [68,69]. Prior to that, a national study carried out in 1863 cats of Northern and Central Italy examined in clinical settings found an average prevalence of 15.3% with 12% of prevalence in Lombardy, and the highest positivity percentages in the northern–eastern area of the country (Friuli-Venezia Giulia 28.7%; Trentino-Alto Adige 21%) and in Tuscany (23.2%). The lowest percentages of positivity were reported in Lazio (7.5%) and Liguria (7.3%) [70]. However, in this study, the cats were tested for diagnostic purposes and most were symptomatic and/or free roaming; therefore, the sampled population was not representative of the feline population in those areas. Nevertheless, a wide distribution and the clinical relevance of FeLV infection were shown at that time in Northern and Central Italy. Older studies from Southern Italy found 7% positivity in Campania [71]. Around the same time and in the following years, no FeLV-positive samples were found in Sicily in studies including stray or outdoor cats [72,73,74,75,76,77], or about 2% positivity was reported in the Sicily [78,79] and Calabria regions [72]. Regional differences in the prevalence of FeLV infection have likely existed for some time within Italy.

One factor contributing to the high FeLV risk of cats in Southern Europe might be the large numbers of stray cats found in these Mediterranean countries that lack harsh winters and have an abundance of food sources. In some Southern European countries, cats are not expected to be owned; they are regarded as natural co-habitants in settlements, and are considered useful for their hunting activity. They are considered not a domestic but a synanthropic species. In some areas, these cats are left unneutered to breed naturally and with limited veterinary support, with serious consequences for the animals and often high mortality of kittens from infectious diseases [80]. In Italy, free-living outdoor cats (gatti liberi) have been protected by law (no-kill no-moving policy) since 1991 [81]. There are registered cat caretakers and compulsory neutering of the cats by the Veterinary Services of the Local Health Unit. This has led to stable cat numbers in Rome [81], but in other Italian regions and other countries, there remain concerns regarding free-roaming cats in general as well as concerning infectious diseases [82]. The no-kill and no-moving restriction has many benefits for the cats, but might be a disadvantage in terms of FeLV infection. Healthy FeLV-positive shedders might stay unrecognized, and so pose an infection risk to uninfected cats. Moreover, if cats are identified as FeLV-infected, they cannot be removed from the cat population.

Amongst the Southern European countries, the FeLV prevalence was lowest in Spain (2.6%). This is lower than reported previously in 2012, when the FeLV infection rate in Barcelona was 6.0% in stray cats [83], and was considerably lower than the 15.6% and 30.4% described in cats taken to veterinarians in the Madrid metropolitan area in 1999 [25]. The low FeLV prevalence in Spain might be associated with the relatively high FeLV vaccination rate in the cats tested from Spain (49.5%) compared to the FeLV vaccination rate in cats from other Southern European countries (Portugal: 14.2%; Croatia: 16.5%; Italy and Malta: 17.8%).

The FeLV prevalence in the Eastern European region was intermediate, between those of the Northern/Western and the Southern European regions, with considerable variation amongst the Eastern European countries. The prevalence was higher in Hungary and Poland (5.9% and 5.0%, respectively). The FeLV-positive cats from Poland were found in the middle and southeastern parts of the country (Figure 1a); a previous study from this region in 2006–2010 had also demonstrated a high FeLV prevalence of 6.4% in clinically healthy cats and cats suspected to have an infectious disease [84], and an even higher prevalence of 14.2% was reported in a study investigating 741 mainly sick cats in the area of Warsaw [85]. Remarkably, none of the 90 tested cats from Bulgaria were FeLV-positive. This could be related to the small number of samples tested or, alternatively, the cats taken to veterinarians in Bulgaria might be well cared for. The FeLV prevalence in the Czech Republic and Slovakia was similar to the overall prevalence in Europe (2.0% and 2.2%) as well as to that reported in a previous study of stray and owned cats in Slovakia [86].

Apart from the site of origin within Europe, other risk factors of FeLV infection were identified in the current study, one being intact males. Tomcats are still considered to be mainly at risk of FIV infection, and male sex has also been described as a risk factor for FeLV infection in other studies [10,14,87,88]. FeLV can no longer be considered only as infection of “social cats”, although FeLV is easily transmitted through social interactions via infectious saliva. However, it is, of course, also spread via the saliva and blood of viraemic cats through aggression, which is a common male behaviour. It is possible that as more cat owners become aware of the fact that FeLV can be transmitted socially and aim to prevent this route of infection (e.g., within a household), the more the transmission by cat fights in cats with outdoor access becomes evident and important. This is supported by the findings that cats exhibiting aggressive behaviour have a higher risk of FeLV infection [14], and cats taken to veterinarians for fighting injuries were frequently FeLV-positive [89]. It is also consistent with the observation here that the outdoor access of the cat was a risk factor for FeLV infection. Cats living outdoors, or having outdoor access at least sometimes, had a significantly higher risk of testing FeLV-positive than cats living indoors. Therefore, it is recommended that all cats with outdoor access should be vaccinated against FeLV in areas/countries where FeLV occurs [90].

Another risk factor identified in this study was the age of the cat. While it was shown previously that young cats are more at risk of developing progressive FeLV infection [24,91], the risk of being FeLV infected in the present study was approximately twice as high in cats aged one to six years compared to younger and older cats. Accordingly, the median age of FeLV viraemic cats was 3 years in a recent study from Germany and 4.75 years in a recent study conducted in the UK. Moreover, in a large study of North American cats tested for FeLV at veterinary facilities and animal shelters, it was observed that adult cats, defined as cats older than 6 months, were more likely to be FeLV viraemic (odds ratio 2.5) than juveniles up to 6 months of age [10]. Thus, FeLV infection should be expected in adult cats and not only in kittens, although the latter have a higher risk of developing progressive infection [91].

As expected, a lower FeLV risk was found in pedigree cats compared to non-pedigree cats: pedigree cats were approximately six to seven times less frequently FeLV-positive than non-pedigree cats. There has been high awareness amongst cat breeders of the risks of FeLV infection for many years, and most cat-breeding facilities are kept FeLV-free; cats are tested as kittens and after every possible exposure risk, and pedigree cats usually have no, or very limited, outdoor access. This further demonstrates that appropriate measures, such as testing, separation, risk assessment/reduction, and if necessary, FeLV vaccination, can significantly reduce FeLV infection within a cat population.

As for other feline viral infections [34,92,93], and in accordance with results from feline retrovirus studies investigating cats in overcrowded conditions [94], keeping cats in groups of ≥5 cats/group was also a risk factor for FeLV infection in the present study. From an epidemiological standpoint, it is generally advisable to keep cats in stable groups that are as small as possible. Moreover, sick cats were more frequently FeLV infected than healthy cats in Europe, which has been described also in other studies [17,32], and has been demonstrated under experimental conditions [5,95]. In contrast to the information on whether a cat was sick or healthy, which was required in the online questionnaire, information on clinical signs was optional and provided voluntarily and not systematically by the attending veterinarians; this represents a limitation of the study. Clinical signs that were associated with FeLV infection in the cats reported in the present study were primarily anaemia, but also anorexia and gingivitis/stomatitis. Anaemia in FeLV-infected cats has been reported previously [96,97,98]. FeLV infection can be associated with different types of anaemia. Pure-red cell aplasia is associated with the rare development of FeLV C within a cat [99], while haemolytic regenerative anaemia is related to opportunistic infections or immune-mediated destruction of red blood cells [98]. However, recent consensus statements suggest that there is only low evidence that FeLV per se induces immune-mediated haemolytic anaemia [100]. FeLV infection is known to lead to anorexia, loss of body weight, and poor body condition [61]. Moreover, in cats with gingivitis/stomatitis, FeLV infection should be considered as an important underlying cause [3].

High FeLV RNA loads (>10^6^ RNA copies/PCR reaction) were measured in the saliva samples of approximately two-thirds of the infected cats and were observed particularly in sick cats. These results cannot be compared directly with the results from virus isolation, although they confirm earlier results of high virus loads shed in infected cats [36,101]. Using RT-qPCR, viral RNA equivalents are measured rather than whole infectious viral particles.

The overall FeLV vaccination rate was rather low (27.1%) in Europe. Although some animals were vaccinated against FeLV in all countries, high variation was found (range of vaccination rate: 3.2%–81.5%). The present study provides unique data on the prevalence of FeLV viraemia and FeLV vaccination rates for many geographic areas of Europe. Remarkably, there are still countries with high FeLV prevalence and low vaccination rates, e.g., Portugal, Italy, Croatia, and Poland. Awareness of FeLV infection and its consequences for the cat and its owner and the protective effect of FeLV vaccines should be increased particularly in these countries, as well as in countries where FeLV prevalence remains at a low level but has not decreased further in recent years.

The results of the present study did not confirm the recently proposed associations between FeLV prevalence and income; it was reported that the highest percentages of FeLV-infected cats lived in areas with lower incomes, whereas a decreasing FeLV infection rate was observed with increasing income [26]. The FeLV prevalence did not correlate with the GDP at PPP in the 30 European countries included in our study. In the present analysis, the data were obtained using the same FeLV detection and cat recruitment method in all countries; in contrast, only limited and/or older data were available from European countries for the recently published meta-analysis, and FeLV prevalence was determined using different methods and heterogeneous study populations [26]. However, the number of countries included in the present study and the range in GDP were somewhat smaller than in the previous study [26], which might have limited the statistical analysis to a degree. Interestingly, we observed a correlation between the FeLV vaccination rate and the GDP at PPP in the investigated European countries. Vaccination rates were higher in areas with higher income and lower in countries/country groups with lower income. This observation can easily be explained since vaccines are more affordable in countries with a higher GDP. A variable vaccination rate might have an indirect effect on the FeLV prevalence. It was noted in the current study that higher FeLV vaccination rates tended to be associated with lower FeLV prevalence rates. However, since this was only a tendency, additional factors other than the FeLV vaccination rate (and the GDP) appear to have an impact on the FeLV prevalence, e.g., the availability of information and awareness concerning FeLV for veterinarians and cat owners.

According to the CART analysis, FeLV-positive cases were grouped into two terminal nodes (Figure 5). The first terminal node 2 corresponds to cats living in Northern and Western Europe (i.e., the area considered at lower risk of FeLV) but living always outdoors (considered a high-risk behaviour). In this terminal node 2, only eight FeLV-positive cats were present within a total of 3511 cats living in Northern or Western Europe. The second terminal node 1 corresponds to cats living in Southern (i.e., area with high prevalence of FeLV) and Eastern Europe (i.e., area with intermediate prevalence of FeLV) but with no pedigree (pedigree cats usually have no or limited outdoor access). Proportionally more FeLV-positive cats were present in this terminal node (i.e., 109 of a total of 2494 cats originating from the southern or eastern parts of Europe), which was expected according to the high to intermediate prevalence of FeLV in these countries in combination with the absence of pedigree cats.

## 5. Conclusions

The low prevalence of FeLV viraemia in Northern Europe and in most of the Western European countries indicates that strict testing and separation programs and vaccination can decrease or even eliminate FeLV infections. A very low FeLV prevalence was generally demonstrated in pedigree cats; cat breeders are aware of FeLV and prevent infection using strict preventive measures. In contrast, in some regions in this study, particularly in Southern Europe, high FeLV prevalence rates were identified, and extra measures will be necessary to control or eliminate FeLV infection from these geographic regions. The present study included countries with significant populations of stray cats and various policies for controlling (or not) these cat populations. While some rescue and shelter cats were included in the study, it can be assumed that the prevalence of FeLV in stray and feral cats that do not attend any veterinary facilities might even be higher in many countries since the living environment is suboptimal, and these cats do not receive any preventive or therapeutic medical care. Moreover, risk factors for FeLV infection, including outdoor access and intact male sex that are commonly associated with aggressive behaviour and fighting, were identified. Thus, in countries where FeLV remains prevalent, cats with outdoor access should be vaccinated and—also for the welfare of the cats—neutered. Awareness of FeLV infection and vaccination should be intensified, particularly in countries with high FeLV prevalence or in countries with lower prevalence but suspected geographic pockets of FeLV-positive cats, e.g., Switzerland and Ireland. In these countries, all cats at risk of FeLV infection should be tested for FeLV. Subsequently, the separation of infected cats and vaccination of uninfected animals is recommended by the Advisory Board on Cat Diseases Europe (ABCD Europe).

## Figures and Tables

**Table 1:** Numbers of veterinarians, samples shipped and returned, and feline leukaemia virus (FeLV) prevalence in the different countries or country groups.

Country or Country Group ^1^	Vets Planned	Participating vets	Samples Shipped	Samples and Data Returned	Return Rate %	No of FeLV-pos. ^2^	FeLV Prevalence % (95% CI) ^3^
**Northern Europe**							
Denmark	40	40	400	277	69.3	0	0.0 (0.0–1.4)
Finland	40	40	400	290	72.5	0	0.0 (0.0–1.3)
Ireland	40	39	390	136	34.9	7	5.1 (2.5–10.2)
Lithuania, Latvia, Estonia	40	40	400	266	66.5	4	1.5 (0.6–3.8)
Lithuania	19	19	190	143	75.3	4	
Latvia	12	12	120	68	56.7	0	
Estonia	9	9	90	55	61.1	0	
Norway	40	38	380	205	53.9	0	0.0 (0.0–1.8)
Sweden	40	40	400	343	85.8	0	0.0 (0.0–1.1)
United Kingdom	40	40	400	136	34.0	1	0.7 (3.8 × 10^−4^–4.0)
England	32	32	320	119	37.1	1	
Scotland	4	4	40	10	25.0	0	
Northern Ireland	2	2	20	0	0.0	0	
Wales	2	2	20	7	35.0	0	
**Eastern Europe**							
Bulgaria, Romania	40	24	240	90	37.5	0	0.0 (0.0–4.1)
Bulgaria	11	11	110	90	81.8	0	
Romania	29	13	130	0	0	n.a.	
Czech Republic	40	40	400	361	90.3	8	2.2 (1.1–4.3)
Hungary	40	40	400	219	54.8	13	5.9 (3.5–9.9)
Poland	40	40	400	340	85.0	17	5.0 (3.1–7.9)
Slovakia	40	40	400	255	63.8	5	2.0 (0.8–4.5)
**Southern Europe**							
Croatia	40	40	400	198	49.5	9	4.5 (2.4–8.4)
Greece	40	0	0	n.a.	n.a.	n.a.	
Portugal	40	40	400	330	82.5	29	8.8 (6.2–12.3)
Spain	40	40	400	352	88.0	9	2.6 (1.4–4.8)
Italy and Malta	40	40	400	349	87.3	20	5.7 (3.7–8.7)
Italy	39	39	390	340	87.2	20	
Malta	1	1	10	9	90.0	0	
**Western Europe**							
Austria	40	40	400	309	77.3	4	1.3 (0.5–3.3)
Belgium, Luxembourg	40	40	400	287	71.8	3	1.0 (0.3–3.0)
Belgium	38	38	380	278	73.2	3	
Luxembourg	2	2	20	9	45.0	0	
France	40	40	400	301	75.3	3	1.0 (0.3–2.9)
Germany	40	40	400	306	76.5	1	0.3 (1.7 × 10^−4^–1.8)
Netherlands	40	40	400	356	89.0	0	0.0 (0.0–1.0)
Switzerland, Liechtenstein	40	40	400	299	74.8	8	2.7 (1.4–5.2)
Switzerland	39	39	390	290	74.4	7	
Liechtenstein	1	1	10	9	90.0	1	
**Total**	920	861	8610	6005	69.7	141	2.3 (2.0–2.8)

^1^ Countries and country groups were classified according to the United Nations geoscheme [29]. As no samples were returned from Romania, it was excluded from prevalence calculations; in Greece, no veterinarians could be recruited, and so it was excluded from all calculations. ^2^ Only results from cats with data in the online questionnaire were included. ^3^ Percentages and 95% confidence intervals (CI) were calculated only for country groups and not for single countries to avoid calculations based on small sample numbers. n.a., not applicable.

**Table 2:** Sample characteristics (all cats and FeLV-viraemic cats).

Variables	Modalities	All Cats (*n* = 6005)	FeLV-Positive Cats (*n* = 141)
Health	Healthy	4060 (67.6 ^1^)	66 (46.8 ^2^)
	Sick	1945 (32.4)	75 (53.2)
Age	<1 year	1826 (30.4)	31 (22.0)
	1 to ≤6 years	2271 (37.8)	80 (56.7)
	>6 years	1908 (31.8)	30 (21.3)
Sex	Female intact	850 (14.2)	15 (10.6)
	Female spayed	1941 (32.3)	38 (27.0)
	Male intact	914 (15.2)	37 (26.2)
	Male castrated	2181 (36.3)	49 (34.8)
	Not sure	119 (2.0)	2 (1.4)
Pedigree	No	5156 (85.9)	139 (98.6)
	Yes	792 (13.2)	2 (1.4)
	Not sure	57 (0.9)	0 (0.0)
Habitat	Private	5151 (85.9)	111 (78.7)
	Breeder	177 (2.9)	0 (0.0)
	Shelter	179 (3.0)	7 (5.0)
	Rescue cat	446 (7.4)	21 (14.9)
	Other	52 (0.9)	2 (1.4)
Multicat environment	Yes	3373 (56.2)	85 (60.3)
	No	2380 (39.6)	44 (31.2)
	Not sure	252 (4.2)	12 (8.5)
Number of cats in group	1	2380 (39.6)	44 (31.2)
	2	1670 (27.8)	32 (22.7)
	3	628 (10.5)	11 (7.8)
	4	295 (4.9)	6 (4.3)
	≥5	708 (11.8)	36 (25.5)
	Not sure	324 (5.4)	12 (8.5)
Access	Indoor only	2193 (36.5)	32 (22.7)
	Indoor and outdoor	3245 (54.0)	83 (58.9)
	Outdoor only	388 (6.5)	23 (16.3)
FeLV vaccination	Yes	1462 (24.3)	14 (9.9)
	No	3938 (65.6)	106 (75.2)
	Not sure	605 (10.1)	21 (14.9)
Last FeLV vaccination	Never	3938 (65.6)	106 (75.2)
	<1 year	943 (15.7)	8 (5.7)
	1 to ≤3 years	337 (5.6)	3 (2.1)
	>3 years	182 (3.0)	3 (2.1)
	Not sure	605 (10.1)	21 (14.9)

^1^ Number in brackets gives the percentage of the number of all cats. ^2^ Number in brackets gives the percentage of the number of FeLV-positive cats.

**Table 3:** Risk and protective factors associated with positive FeLV viraemic status of cats after univariate logistic regression.

Variables	Modalities	FeLV Prevalence	95% Confidence Interval ^a^	Odds Ratio	95% Confidence Interval ^b^	*p*-value
Europe	Eastern	3.4	2.5–4.6	Reference	-	-
	Northern	0.7	0.4–1.7	0.21	(0.11–0.40)	<0.001 *
	Southern	5.5	4.2–6.9	1.64	(1.11–2.42)	0.013 *
	Western	1.0	0.6–1.6	0.29	(0.17–0.51)	<0.001 *
Pedigree	No	2.7	2.2–3.1	Reference	-	-
	Yes	0.3	0.0–1.0	0.09	(0.02–0.37)	0.001 *
Habitat	Breeder	0.0	0.0–2.1	Reference	-	-
	Rescue cat	4.7	2.8–6.8	17.94	(1.08–297.85)	0.044 *
	Private	2.2	1.7–2.5	17.57	(0.82–372.10)	0.07
	Shelter	3.9	1.5–7.6	7.85	(0.49–126.89)	0.15
	Other	3.8	5.0–13.2	15.43	(0.87–272.42)	0.06
Number of cats in group	1	2.1	1.6–2.7	Reference	-	-
	2	1.9	1.3–2.7	0.92	(0.60–1.43)	0.72
	3	1.8	0.9–3.1	0.84	(0.44–1.62)	0.61
	4	2.0	0.7–4.4	0.98	(0.42–2.30)	0.97
	≥5	5.1	3.6–7.0	2.53	(1.65–3.88)	<0.001 *
Sex	Female intact	1.8	1.0–2.9	Reference	-	-
	Female spayed	2.0	1.4–2.7	1.11	(0.61–2.03)	0.73
	Male intact	4.0	2.9–5.5	2.35	(1.28–4.31)	0.006 *
	Male castrated	2.2	1.7–3.0	1.28	(0.71–2.29)	0.41
	Not sure	1.7	0.2–5.9	0.95	(0.21–4.21)	0.95
Age	< 1 year	1.7	1.2–2.4	Reference	-	-
	1 to ≤6 years	3.5	2.8–4.4	2.11	(1.39–3.22)	<0.001 *
	>6 years	1.6	1.1–2.2	0.92	(0.56–1.53)	0.77
Access	Indoor only	1.5	1.0–2.1	Reference	-	-
	In- and outdoor	2.6	2.0–3.1	1.77	(1.17–2.67)	0.006 *
	Outdoor only	5.9	3.8–8.8	4.26	(2.46–7.35)	0.001 *
	Not sure	1.7	0.3–4.8	1.15	(0.35–3.80)	0.82
Last FeLV vaccination	Never	2.7	2.2–3.2	Reference	-	-
	<1 year	0.8	0.4–1.7	0.31	(0.15–0.64)	0.001 *
	1 to ≤3 years	0.9	0.2–2.6	0.32	(0.10–1.03)	0.056
	>3 years	1.6	0.3–4.7	0.61	(0.19–1.93)	0.40
	Not sure	3.5	2.2–5.3	1.30	(0.81–2.09)	0.28
Health	Healthy	1.6	1.3–2.1	Reference	-	-
	Sick	3.9	3.0–4.8	2.43	(1.74–3.39)	<0.001 *

^a^ Confidence interval for the mean; ^b^ confidence interval for the odds ratio; * *p*-value < 0.05.

**Table 4:** Risk and protective factors associated with the feline leukaemia virus (FeLV) viraemia of cats after multivariate analysis.

Variables	Modalities	FeLV Prevalence	95% Confidence Interval ^a^	Odds Ratio	95% Confidence Interval ^b^	*p*-value
Europe	Eastern	3.4	2.5–4.6	Reference	-	-
	Northern	0.7	0.4–1.7	0.29	(0.15–0.56)	<0.001 *
	Southern	5.5	4.2–6.9	1.81	(1.20–2.72)	0.005 *
	Western	1.0	0.6–1.6	0.42	(0.23–0.74)	0.003 *
Pedigree	No	2.7	2.2–3.1	Reference	-	-
	Yes	0.3	0.0–1.0	0.15	(0.04–0.60)	0.008 *
Number of cats in group	1	2.1	1.6–2.7	Reference	-	
	2	1.9	1.3–2.7	0.96	(0.62–1.51)	0.87
	3	1.8	0.9–3.1	0.79	(0.41–1.54)	0.49
	4	2.0	0.7–4.4	0.90	(0.39–2.15)	0.82
	≥5	5.1	3.6–7.0	1.63	(1.03–2.58)	0.040 *
Sex	Female intact	1.8	1.0–2.9	Reference	-	-
	Female spayed	2.0	1.4–2.7	1.38	(0.72–2.64)	0.33
	Male intact	4.0	2.9–5.5	2.24	(1.20–4.18)	0.01 *
	Male castrated	2.2	1.7–3.0	1.48	(0.79–2.78)	0.23
	Not sure	1.7	0.2–5.9	0.97	(0.21–4.53)	0.84
Age category	< 1 year	1.7	1.2–2.4	Reference	-	-
	1 to ≤6 years	3.5	2.8–4.4	2.04	(1.27–3.28)	0.003 *
	>6 years	1.6	1.1–2.2	1.01	(0.56–1.83)	0.97
Access	Indoor only	1.5	1.0–2.1	Reference	-	-
	In- and outdoor	2.6	2.0–3.1	1.72	(1.12–2.65)	0.01 *
	Outdoor only	5.9	3.8–8.8	1.88	(1.03–3.44)	0.04 *
	Not sure	1.7	0.3–4.8	1.07	(0.31–3.69)	0.92
Last FeLV vaccination	Never	2.7	2.2–3.2	Reference	-	-
	<1 year	0.8	0.4–1.7	0.49	(0.23–1.03)	0.06
	1 to ≤3 years	0.9	0.2–2.6	0.39	(0.12–1.26)	0.11
	>3 years	1.6	0.3–4.7	0.79	(0.24–2.62)	0.70
	Not sure	3.5	2.2–5.3	1.40	(0.85–2.33)	0.19
Health	Healthy	1.6	1.3–2.1	Reference	-	-
	Sick	3.9	3.0–4.8	2.04	(1.41–2.90)	<0.001 *

^a^ Confidence interval for the mean; ^b^ confidence interval for the odds ratio; * *p*-value < 0.05.

**Table 5:** Major clinical problems reported in FeLV viraemic sick cats in comparison with all sick cats.

Clinical Problem	FeLV-Negative Sick Cats (%)	FeLV-Positive Sick Cats (%)	Odds Ratio (95% Confidence Interval)	*p*_F_ *
Anaemia Yes No	23 (1.2) 1847 (98.8)	8 (10.7) 67 (89.3)	9.6 (4.4–21.4)	<0.0001
Anorexia Yes No	45 (2.4) 1825 (97.6)	6 (8.0) 69 (92.0)	3.5 (1.6–8.3)	0.0122
Gingivitis and/or stomatitis Yes No	121 (6.5) 1749 (93.5)	11 (14.7) 64 (85.3)	2.5 (1.3–4.8)	0.0152

* *p*_F_: *p*-value Fisher’s exact test.

## Appendix A

**Table A1:** Online Questionnaire.

Parameter	Type	Value Range	Value Required
Country identification	select	list of countries	yes
Veterinary practice	text		yes
Six-digit swab number	text	according to tube label	yes
Date of collection	date		yes
Cat name	text		yes
Owner name	text		yes
Postal code of cat owner	text		yes
Type of husbandry	select	private/breeder/shelter/rescue cat/other	yes
Multicat environment	select	yes/no/not sure	yes
If yes: how many cats in the group	select	2/3/4/≥5	optional
Sex of cat	select	male/female/not sure	yes
Reproductive status of cat	select	intact/neutered/not sure	yes
Age of cat	select	0–8 weeks/9–12 weeks/13–52 weeks/1–2 years/2–3 years/…/19–20 years/≥20 years	yes
Pedigree cat	select	yes/no/not sure	yes
If yes: which breed	text		optional
Indoor and outdoor access	select	Indoor and outdoor/indoor only/outdoor only/not sure	yes
Last vaccination against FeLV	select	<1 year/1–3 years/>3 years/never/not sure	yes
Health status	select	healthy/sick	yes
If sick: major clinical problem	text		optional
Comments	long text		optional
